# Panoramic view of diversity and function of cuticular proteins in insects and mosquitoes biology

**DOI:** 10.3389/finsc.2025.1602055

**Published:** 2025-08-29

**Authors:** Yamini Thakur, Sanjay Tevatiya, Gaurav Kumar, Meenakshi Jeena, Vaishali Verma, Rajnikant Dixit, Shweta Pasi, Alex Eapen, Jaspreet Kaur

**Affiliations:** ^1^ Department of Vector Genomics and Vector Biology, ICMR- National Institute of Malaria Research, New Delhi, India; ^2^ Academy of Scientific and Innovative Research (AcSIR), Ghaziabad, India; ^3^ ICMR-National Institute of Nutrition, Hyderabad, Telangana, India; ^4^ ICMR-National Institute of Malaria Research, Field Unit, Chennai, India

**Keywords:** insect, mosquitoes, cuticle proteins, cuticle genes, protein domain, anopheles

## Abstract

**Aim:**

The insect cuticle, vital for structural maintenance, forms their exoskeleton. It is mainly composed of an intermesh of – structural cuticle proteins (CPs) with polysaccharide chitin. The insect CPs encoded by *CP* genes are indispensable for morphology, development and adaptation to various ecological niches across all life stages. The number of CPs may vary across genera and species, with almost 150 proteins in *Bombyx mori* and more than 298 CPs found in *Anopheles gambiae*. While they have been extensively studied in insects such as agricultural pests, limited studies have been conducted on mosquitoes, particularly those relevant to public health, such as the *Anopheles* a key malaria vector.

**Objective:**

This review recapitulates current knowledge on CPs in insects, while also underscoring vital knowledge gaps regarding regulation and metabolic crosstalk of CPs with other signaling and/or metabolic pathways.

**Methods:**

We performed a comprehensive review of published studies and extracted data from databases including Vectorbase and NCBI with the aim of retrieving information on cuticular proteins, their gene families, abundance and associated functions. Additionally, we identified and analyzed the gaps in the available information. A literature search was conducted between (2000 and 2025) in an electronic database using PubMed, Scopus and Google Scholar. The search keywords were: cuticular proteins, cuticular genes, *Anopheles*, mosquito cuticle proteins, insecticide resistance, and CP gene families.Inclusion criteria: peer-reviewed research articles and review papers particularly focused on CPs in insects and *Anopheles* mosquito species.

**Results:**

In the present review, we provide comprehensive analysis of cuticle protein families across insects including mosquitoes based on available data. We further highlight their basic constituents and protein domain structure, offering insight into their role in insect physiology. We have effectively integrated insect studies with mosquito-specific research on CPs (bridging the gap between insect and mosquito-specific research). This holistic approach would facilitate a broader comprehension of CPs in both insect and mosquito vectors.

**Main conclusions:**

The goal of this study is to enhance our understanding of insects and *Anopheles* biology and how studies on CPs could be leveraged to develop novel strategy for management of pest and combat vector-borne diseases (VBDs).

## Graphical Abstract

The figure illustrates the wide-ranging functional roles of cuticular proteins (CPs) in insects and mosquito vectors. Each panel showcases a distinct biological function (1) structural development (2) insecticide resistance, (3) mating behavior, and (4) metamorphic development.

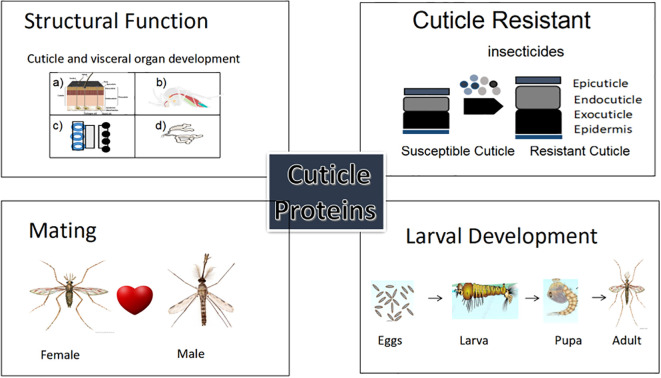

## 1 Introduction

The Phylum Arthropoda, the largest phylum under metazoa, encompasses the class Insecta, characterized by distinct features such as hard exoskeletons, chitin, and jointed appendages (1). Insects are renowned for playing crucial roles in ecosystems such as decomposing organic substances, participating in predator-prey and host-parasite interaction as well as maintaining ecological balances, however they also represent significant threat to public health and the agricultural industry (2). The cuticle of insect is central to their success, playing a multitude of roles essential for their survival. The insect cuticle offers a broad range of function including protection against water desiccation and retention, providing immunity, aid to locomotory movement and adhesion, facilitate morphogenetic shedding of the cuticle during their life cycle development, and temporary storage of food during starving or any unfavorable condition (3, 4). These research studies underscore the importance of CPs in insect biology. Targeting cuticular proteins offers a promising approach for developing novel insecticides.

The cuticle of insects consists of two primary layers: the epicuticle (without chitin) and the procuticle. The epicuticle is further subdivided into the outer epicuticle and the inner epicuticle (5). The outer layer of the epicuticle, cuticulin, consist of various protective layers, including a tanned thin layer made up of polyphenols and lipids whereas the inner epicuticle comprises lipids and proteins that form a barrier, rendering insects impermeable to water, preventing from desiccation and enzymatic digestion. Additionally, this layer facilitates the gradual release of hydrocarbon compounds (pheromones) during mate and colony selection (6). The inner layer of the cuticle, the procuticle, is further divided into the exocuticle and the endocuticle. The exocuticle, the outermost layer, also referred to as the envelope of the membrane, forms a physically harder layer. The endocuticle, formed by softer poly-N-acetylglucosamine (chitin), is composed of characteristically distinct cuticular proteins (CPs) known as arthropodin (7, 8). The CPs and chitin filaments act as functional partners to facilitate the shedding of old cuticles and the formation of new cuticle during the molting process.

The insect cuticle, the outermost complex and essential protective exoskeleton layer, varies in composition among genera and species (9). Furthermore, its composition alters at every metamorphic stage of insects (10). For instance, the cuticular layer of the *Drosophila melanogaste*r undergoes alteration during its various development stages accordingly (11). The process of pigmentation and sclerotization eventuates as the cuticle ages wherein CPs are cross-linked to chitin filaments through quinones or quinone methides produced by laccase -2 enzyme, which oxidizes N-acetyl catechols (12). During sclerotization, CPs participate in the formation of histidine-β-dopamine, the primary adduct of hard cuticles, which is formed by the histidine amino acid residues in CPs (13). CPs, encoded by *CP* genes, make up approximately 1–2% of an insect’s genome (14). Consequently, these proteins offer diverse functionalities tailored to each developmental stage of the insect (5, 11, 15–17). The world incurs heavy economic losses due to insect pests which also jeopardize food security (18). Additionally, mosquitoes through transmission of parasites and pathogens cause several VBDs, significantly contributing to morbidity and mortality in public across the globe (According to 19). Given that the CPs play vital roles at each developmental stage of the insects including mosquito, from the larval to the adult stage- and some are even activated in the egg stage. The study of the CPs is crucial for comprehending the life cycle, morphology, and physiology of insects. Evaluating the mechanistic insight into the role of CPs can empower us to comprehend the underlying molecular mechanisms governing the insect/mosquito physiology and may open a new avenue in discovering novel targeted pest and vector control tools.

## 2 Cuticle protein

**Figure 1:**
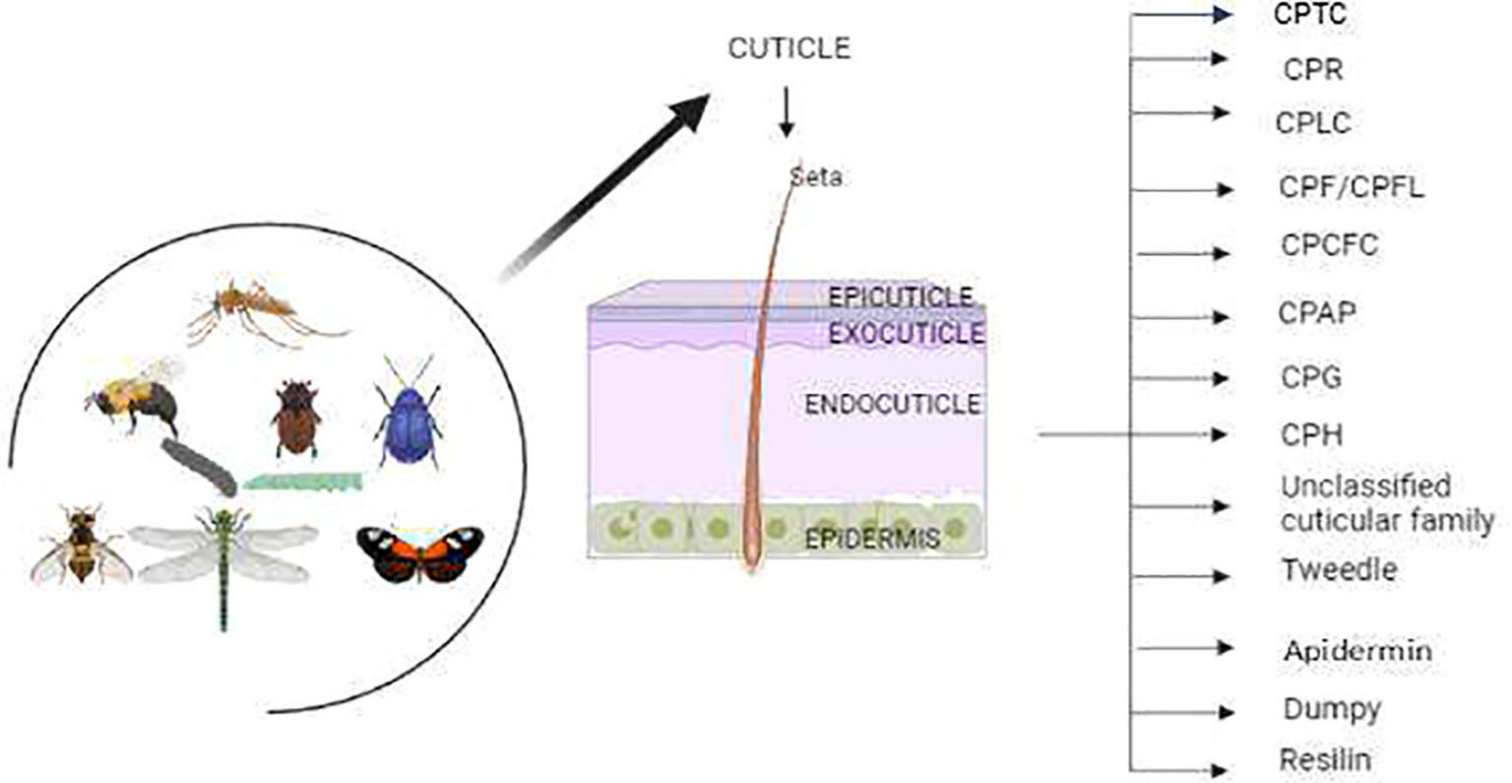
The insects have two major cuticle layers 1) Exocuticle and 2) Procuticle, where procuticle layers are subdivided into epicuticle and exocuticle. The Procuticle layers have 13 major cuticle proteins in insects including moths, beetles, flies and mosquitoes.

The CPs have been characterized with unique protein motifs, signal peptides, and a chitin-binding domain. Nearly every CP has glycine, proline, and tyrosine amino acid residues with distinct features and functions. Overall, the CPs have been categorized into different families based on their conserved motifs (**Table 1**). Certain protein families are unique to specific insect groups (**Figure 1**), such as the CPLC family found in *Dipterans* while others are exclusive to particular insect species, like Apidermins in honey bees and Dumpy in fruit flies (26, 33).

**Table 1:** The cuticular proteins family is divided into 13 major groups (as mentioned in **Figure 1**).

S. no.	Cuticle proteins family	Authors
1.	CPR Family (Rebers and Riddifods domain family)	(20, 21)
2.	CPF/CPFL (Cuticular Protein with 44 consensus amino acids)	(22, 23)
3.	CPAP (Cuticular Proteins Analogous to Peritrophins)	(24, 25)
4.	CPLC (Cuticular Protein of Low Complexity)	(26)
5.	Unclassified low-complexity Cuticular protein	(26)
6.	CPCFC (Cuticle Protein with two Cysteines separated by the Five amino acids)	(27, 28)
7.	CPG Family (Cuticular Protein Glycine-rich)	(29, 30)
8.	CPH (Cuticle Protein Hypothetical)	(31)
9.	Tweedle	(32, 33)
10.	CPTC (Cuticular Proteins family with two Cysteine residues)	22)
11.	Apidermin	(33, 34)
12.	Dumpy	(35)
13.	Resilin	(36)

## 3 Common domain structure of the Cuticle Protein

**Figure 2:**
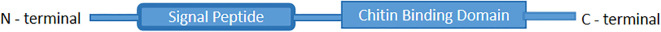
General diagram of cuticle proteins having signal peptides at the N – terminal and at least a chitin-binding domain in peptide.

The cuticular protein is a large family of proteins that also have various common amino acid residues and motifs in their peptides. The occurrence of the motifs depends on the function of the peptides (33, 37, 38). In many CP families, various cuticular genes are found to have a single signal peptide (158/160) at the N-terminus (mentioned in **Figure 2**), repeats of GGX or AAP (AV), and at least one chitin-binding domain as described in **Figure 2**, except the *CPF/CPFL* gene family (13, 38, 39). The cuticle proteins are normally secreted by epidermal cells and categorized into protein families based on their conserved motifs (40).

## 4 Cuticular protein families

### 4.1 CPR family

**Figure 3:**
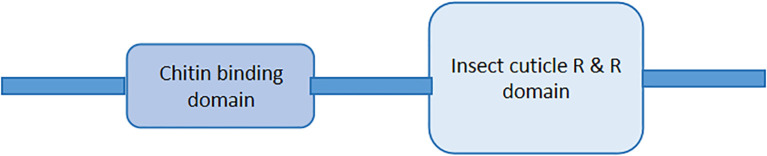
The RR domain structure of all three CPR proteins - The CPR family have a chitin-binding domain at the C-terminal and one Conserved RR - domain at the N-terminal.

CPR family represent the Chitin-binding type R&R consensus with the Interpro ID: IPR031311 (https://www.ebi.ac.uk/interpro/search/text/IPR031311, Pfam ID PF00379). It is the largest cuticular family. This family derives its name from the discoverers of the RR motif- Rebers and Riddiford (20, 21). The RR domain possesses 35–36 amino acid consensus sequences and is present in the cuticular layers of the insects (**Table 2**). The extended consensus sequence having 53 amino acids is critical for chitin binding. All the RR family proteins have an RR-2 domain with the distinct sequence GFNAVV in the C-terminal of the peptide (37, 42) (refer to **Figure 3**). The family divides into two major sub families i.e., RR-1 and RR-2. RR-1 is present exclusively in exocuticle while RR-2 is present in both the endocuticle and exocuticle and is more histidine rich compared to RR-1 (39, 41). The conserved sequence (34–36 amino acids) extended to the 65 amino acids long stretch and was further divided into the three families, RR-1, RR-2 and RR-3. RR-3 is a small sub family, and the distinct features of this family of CPs have not been defined (44). The RR family has a high amount of histidine residues which are functionally supposed to facilitate cuticle-hardening (13).

**Table 2:** The CPR family has three different types of domains, differentiated by each group’s characteristics.

Characteristics	RR1 FAMILY	RR 2 FAMILY	RR3 FAMILY	Author
Amino acid residues (Consensus)	58 amino acid residue motifs (most evolving)	68 amino acid residue motifs (conserved consensus is high for the other two families)	75 amino acid residue motifs	(39, 41)
Localization	Exocuticle, except CPR151	Endocuticle and exocuticle	not defined yet	(39, 41, 42)
Stages (Morphologically)	Consistency of expression is unstable. Some genes are expressed in L4, but they are not present in the pupae or adult pharate.	Consistency throughout all stages, from L1 to adult Pupae	Post ecdysal (not confirmed)	(39, 43)
Histidine rich residues	Absent	Present	Not reported Yet	(13)

#### 4.1.1 CPR functions

In insects, CPs undergo repeated cycles of formation and degradation, essential for their transition from larval stages to adult stage, in the molting process. The *CPR* genes, accountable for cuticle formation, are significantly expressed in *Tetranychus urticae* possibly under the regulatory network of juvenile hormone and ecdysterone hormone dynamic during the development process (15, 45). RR1, a subtype of CPR, is remarkably associated with flexible and less sclerotized cuticle regions (for example, hind wings and intersegmental membrane) while RR2 subtype is related to rigid cuticle regions (for example, head capsules (14, 46). The functional studies based on the RR-3 subfamily have not yet been reported (44),

Many studies have evidenced that the *CPR* genes (e.g. CPR63 and CPR47*)*, expressed in the exocuticle as well as endocuticle segment of the cuticle layer in insects, attribute to resistance against insecticide and confer protection from the hostile environmental during unfavorable conditions (43, 47, 48). Research on *Tribolium castaneum* suggests that the *TcCPR4* gene is responsible for the rigidity of the cuticle and that silencing of this gene results in malfunctioning in pharate adults (12). One of the study also highlighted that transcripts corresponding to RR-2 proteins were widely distributed and highly expressed in the ovary of the *Bombyx mori* (29).

### 4.2 CPF/CPFL

The family have 44 conserved amino acid motifs and a C-terminal motif at the end. The reason CPFL is named CPFL-like is that the protein group shares the same C-terminal motif after the sequence but lacks exact homology with a 44 amino acid motifs. The conserved regions contain three aromatic amino acids, and the two families do not bind with chitin (22, 23, 39). The Pfam and InterPro IDs are PF11018 (https://www.ebi.ac.uk/interpro/entry/pfam/PF11018) /IPR022727 (https://www.ebi.ac.uk/interpro/entry/InterPro/IPR022727). Unlike other CPs, the CPF/CPFL Family proteins lack a chitin-binding domain, demonstrating that these proteins may not be associated directly with chitin. Alternatively, they may confer a non- structural role or any auxiliary role in insect cuticle.

According to (23), the CPFL expression was higher during molting of the larvae. The mRNA of *CPFL* genes 2–7 are significantly upregulated just after ecdysis in *Anopheles gambiae* mosquito (**Table 3**).

**Table 3:** The expression pattern of the CPF and CPFL an insect’s life cycle.

Stages	CPF	CPFL	Reference
Eggs	Present, low	Absent	(23, 49)
Larvae	Present, low	Absent	(23, 49)
Pupa	Absent	Present, high	(23, 49)
Adult	Present, low	Absent	(23, 49)

#### 4.2.1 Function of CPF/CPFL

Despite the presence across all insects, the function of CPF/CPFL proteins is still unclear. These proteins lack the chitin-binding domain and are not associated with chitin within the cuticle. However, their gene expression pattern was shown very active during the larval-to-adult transition, possibly underscoring their functional role in the molting process (39, 50).

### 4.3 CPAP family

**Figure 4:**
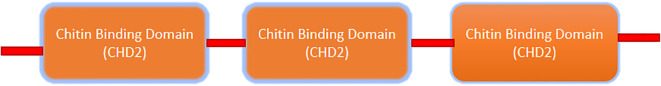
The common Domain structure of the CPAP family has three chitin-binding domains (https://www.ebi.ac.uk/interpro/result/InterProScan/iprscan5-R20231117-095218-0179-21412921-p1m/).

The CPAP family comprises the three chitin-binding domains (CHBD2) with conserved cysteine residues, initially identified as obstructor protein (obsA) in *Drosophila melanogaster* (refer to **Figure 4**) (24, 51). Later, the protein family was renamed as the CPAP family characterized by having six cysteine residue rich CHBD2 domains (24, 25, 51). Overall, the *CPAP* genes, based on the number of CHBD2 they contain, are categorized into two families CPAP1 (one domain) and CPAP3 (three domains).

#### 4.3.1 CPAP function

The *CPAP* genes are primarily localized to and expressed in tissue derived from ectoderm including the epidermis, trachea, hindgut, and foregut (24, 39). These genes are primarily known for scaffolding the cuticle structure (39), The Obs-A protein, belonging to the CPAP3 family, interacts with proteins such as, Knickkopf, Serpentine (chitin deacetylase) and chitin within the cuticle extracellular matrix (ECM) to form a complex, termed as obs-A complex. This complex reinforces the cuticle ECM and shields the cuticle from digestion and premature degradation during development (52). Silencing of *CPAP1* and *CPAP3* genes in *T. casteneum* results in abnormalities in the development stages, and specifically silencing of the *CPAP3* genes lead to abnormal development of wings, walking defects, and increased post-eclosion adult mortality (53). One of the study also reported their possible role in cuticle resistance mechanism. The *CPAP3* genes (*CPAP3-A1*, *CPAP3-C1*, *CPAP3-D1*, and *CPAP3-E2*) expression increased significantly in *Bactrocera dorsali* when exposed to malathion (54).

### 4.4 CPLC

The family includes the CPLCG, CPLCW, CPLCP, and CPLCA families (**Table 4**). The CPLC stands for the high proportion of low complexity sequence, and the last suffix stands for the single letter of each family’s most repeated amino acid (26).

**Table 4:** The CPLC subgroups and their amino acid-rich content with class insects orders.

Gene	Amino acid rich content	Order	Author
*CPLCG*	Two invariant glycine in conserved region	Diptera	(13, 26)
*CPLCA*	Alanine-rich residues in conserved regions	Diptera, Lepidoptera	(26, 49)
*CPLCP*	Proline rich residues in conserved regions	Diptera, Lepidoptera	(13, 26, 55)
*CPLCW*	Tryptophan rich residues in between the conserved domain	Mosquitoes	(13, 26)

#### 4.4.1 CPLCG

The first proteins of the gene family were identified as *Dacp-1* and *Dacp-2 in Drosophila melanogaster* (11). The suffix ‘G’ at the end of the CPLCG denotes the two invariant glycine residues in the conserved region separated by a few amino acids and are considered to be exclusive to the order *Diptera* (13, 26).

#### 4.4.2 CPLCA

The subfamily named as the CPs consists of two or more conserved alanine residues in their conserved region. The first two members of the gene were found by He et al. (22) in *An. gambiae*. The *An. gambiae* consists a conserved homologous sequences of the retinin domain for CPLCA proteins (13, 26). (13, 26). The gene family members are also identified in *Lepidoptera* (for example, *Bombyx mori and Dendrolimus punctatus*) (49).

#### 4.4.3 CPLCP

Cornman and Willis (26) described the CPLCP gene family as enriched with proline. The four members of this family have been found in *Drosophila*, which encodes proline, lysine, valine, and tyrosine. However, in recent years, CPLCP has also been discovered in *B. mori* (55, 56).

#### 4.4.4 CPLCW

The protein is rich in tryptophan, and the gene family is exclusively expressed only in mosquitoes; CPLCW refers to an invariant tryptophan within the conserved domain (13, 26).

#### 4.4.5 Function of the *CPLC* gene

*CPLC* gene has been reported to contribute to insecticide resistance (IR) in different genera of mosquitoes. It has been found that the CPLCG subgroup has been particularly conferring IR in *Culex* and *Anopheles* mosquitoes, by facilitating cuticle thickening and thereby preventing cuticle penetration. However, the roles of other subgroups of the CPLC Family in the IR mechanism remain unknown. The reduced expression of CPLC genes along with *CPFs* and *CPR* genes has been shown to delay ecdysis and cause deformities in adult mosquitoes (57).

### 4.5 Unclassified low-complexity Cuticular protein

The Unclassified low-complexity Cuticular protein, initially discovered in *An. gambiae* by Cornman and Willis (26) and Willis (33), is also a low-complexity sequence but is not assigned to any CPLC family. The Cornnan named this family “CPLCX’’ as it corresponds to the chromosome number at which they have expressed. They are rich in alanine, proline, and tyrosine, and they almost have the same length and amino acid composition (26). The function is not known, but the family is exclusive to mosquitoes, particularly *An. gambiae* (26).

### 4.6 CPCFC family

Jensen et al. (27) discovered the first CPCFC gene in the cuticle of the cockroach (*Blaberus craniifer*) in the nymph stage as *Bc-NCP1.* The CPCFC name was designated by Willis et al. (28). The name CPCFC was described as a protein that has a motif with two cysteines separated by the five amino acids, i.e., C-X(5)-C, and these motifs are repeated by three times within16 amino acids (27, 28, 58).

#### 4.6.1 Function of CPCFC

Experimental data from *An. gambiae* shows that the expression of *agamCPCFC1* transcripts increases immediately following molting. The study revealed that CPCFC1 protein is transferred to the endocuticle where it promotes cuticle hardening (58). In *Manduca sexta*, the *CPCFC* gene has been found in the head of post-molt larvae but is absent in adults (59).

### 4.7 CPG family

Glycine-rich protein family with conserved sequence repeats of GXGX or GGXG named as Ld-GRPs in *Lepidoptera* found in epidermal cells or epidermis (16, 29, 30, 33). The first *CPG* gene was discovered by Suzuki et al. (60) as CPG1, and Futahashi et al. (29) found 29 whole new *CPG* genes in *B. mori* and named them CPG (cuticular protein glycine-rich).

#### 4.7.1 Function of *CPG* genes

The *CPG* genes are richly expressed during pupal stages. In *B. mori*, transcript of *CPG* genes have been reported to be present in the hard cuticle structure including the pheromone gland, compound eye, and maxillary galea (29).

During the insect molting process, the cuticle layer undergoes a cuticle renewal process (repeated cycles of synthesis and degradation) managed by the coordinated action of ecdysteroid and juvenile hormone (JH). Suzuki et al. (60) reported that transient surge of ecdysteroid hormone during molt induces heightened expression of CPG in epidermis in *B. mori.* The protein, encoded by CPG1, serves as important constituent of epicuticle and is possibly implicated in cross-linking by its GGY repeats.

### 4.8 Apidermin

This *CP*s gene family is exclusive to the *Apis* genus and was first identified in the *Apis mellifera.* The family consists of three hydrophobic proteins distinguished by the high alanine content (33, 34).

#### 4.8.1 Function of apidermins

The spatial-temporal analysis of *apidermin* gene family proteins revealed different expression patterns in different tissues. *Apd-1* gene was predominantly expressed in the epidermis underlying the cuticle layer possibly bound for sclerotization whereas *apd-2* as well as *apd-3* genes were expressed in the trachea and digestive tract. *Apd-3* gene was also found to be expressed in the outer epidermal structure such as eyes (33).

### 4.9 CPH

The protein family was also discovered by Futahashi et al. (29) in *B. mori* and designated a group of 34 proteins as cuticular protein hypothetical (CPH) characterized by a signal peptide and possessing sequence similarity to known *CPs* gene. Some of the family members are characterized by the presence of AAP (A/V) motifs specific to cuticle protein genes (31). The members of these families are highly active during the larval stage (61).

#### 4.9.1 The function of CPH

The CPH plays a major role in the larval molting process, contributing to the cuticle thickening and conferring resistance to deltamethrin in *Lepidopterans* larvae (61). Gan et al. (62) discovered a midgut-specific *CPH* like gene in *B.mori* which plays a vital role in histogenesis.

### 4.10 Tweedle family

**Figure 5:**
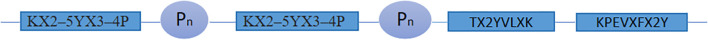
The blocks and their arrangements with proline-rich amino acids in the tweedle protein.

The *Tweedle* gene was named after its discovery in the mutant Drosophila TweedleD, which showed drastic changes in body shape and size in the larval stage and resembled the corpulent Tweedledee (short and stout) (32, 33). Guan et al. (32) proposed that the observed body alteration is due to the *Tweedle* gene and is associated with changes in the cuticle. The tweedle gene is thought to have evolved 300 million years ago through insects (26, 33). The tweedle protein does not have RR-conserved sequences. They have aromatic acids that facilitate the formation of bonds with chitin in the ECM. Tweedle has four conserved blocks, and the tweedle motif is made up of a beta-barrel structure of multiple beta-strands, which are necessary for the binding of the cuticle protein with chitin (32, 63). The conserved blocks consist of (a) the consensus of KX2–5YX3–4P in Blocks 1 and 2; (b) the consensus of Block 3 is TX2YVLXK; and (c) the consensus of Block 4 is KPEVXFX2Y, which is most conserved among insects, and the amino acids separated by blocks 1, 2, and 3, 4 are variable and proline-rich among insects and then there is abundance of proline in block 1 and 2 also (X represents the non-conserved amino acid residues within the consensus region site) (mentioned in **Figure 5**) (64).

#### 4.10.1 The function of the tweedle family

It has been discovered that members of this CP family significantly impact insect cuticle development and their molting process. It is evident from the study demonstrated in *Locusta migratoria* where silencing of the *LmTwdl1* gene, belonging to the tweedle family, led to high mortality before being molted to the next phase and thinner cuticle development (65). Soares et al. (66) studied the cross-talk of ecdysteroid hormones with *tweedle* genes in honey bees and their expression was found to be higher in the outer integument after ecdysteroid peak during adult exoskeleton development. Separately, heightened expression of the tweedle gene in the epidermis of *Drosophila* and *B.mori* also pinpoints their role in cuticle development. Apart from cuticle formation, higher expression of *tweedle* genes in the gut tissue of honey bees underscore their possible role in the formation of PM (peritrophic matrix). In a recent finding, Wang et al. (67) investigated the molecular factor conferring tolerance to exposed cold and hypoxia in *Drosophila suzukii* larvae. The study revealed that expression of structural cuticle genes, especially from the tweedle family, along with ATP-dependent proton pump provided biological adaptation to withstand cold and hypoxic conditions.

### 4.11 Dumpy

**Figure 6:**
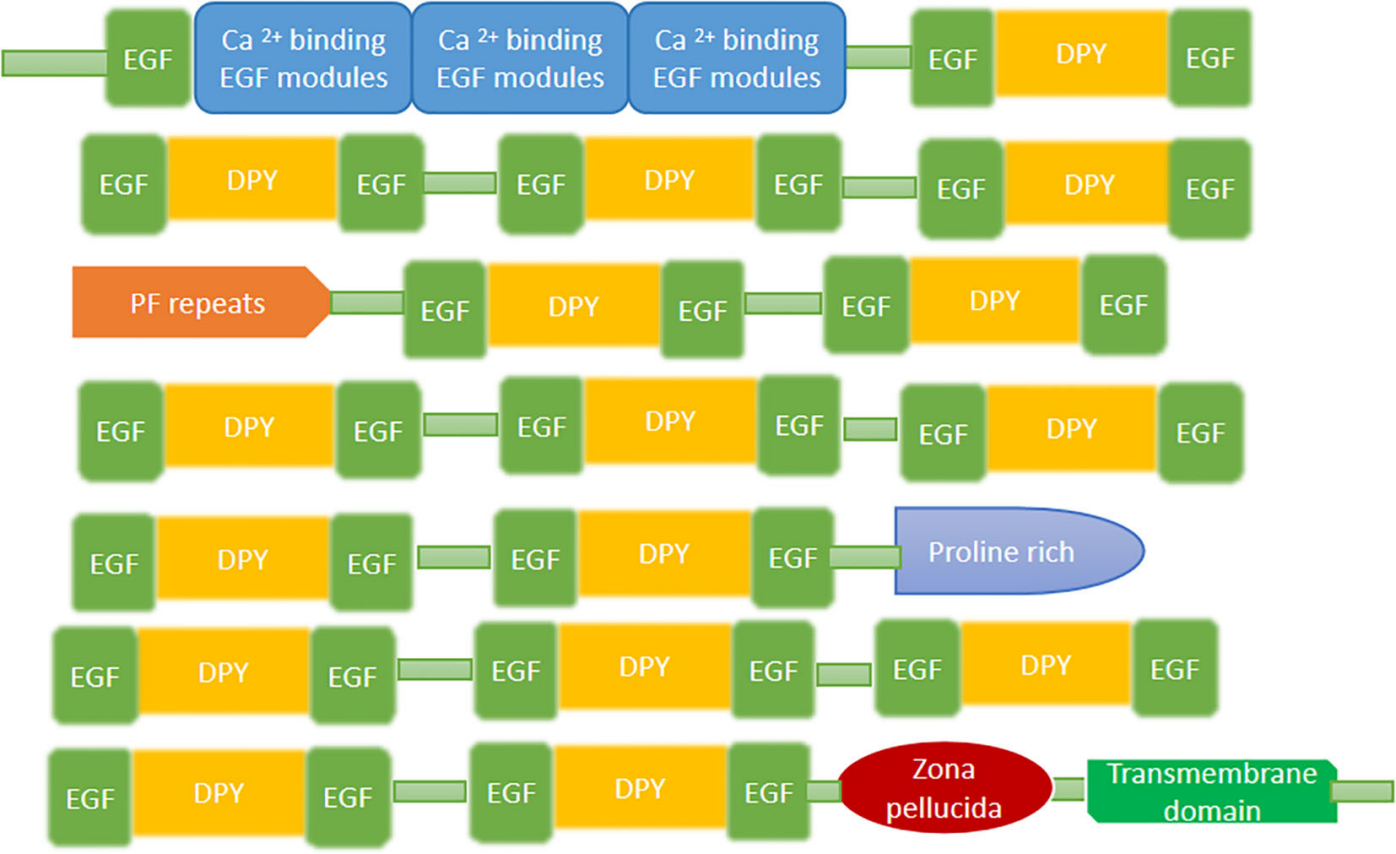
The domain structure of the dumpy protein found in the *An. gambiae*.

The Dumpy protein, characterized by having cysteine residue in its conserved region, was identified in *Drosophila melanogaster* and it is one of the largest protein found in *D. melanogaster*. The dumpy (DPY) protein is found in the ECM with EGF factors in its structure (35). The structure contains the multiple EGF-DPY-EGF triad domains; DPY has the cysteine-spacing pattern, with two repeated regions containing conserved tandem repeats of serine/threonine-rich P-F repeat (PF) and proline-rich repeats altogether with the PGVINIPSVPQP consensus motif. The N-terminus is enriched in calcium-binding EGF repeats, and the C-terminus contains a transmembrane domain and a Zona Pellucida (ZP) domain (mentioned in **Figure 6**) (35, 68).

#### 4.11.1 The function of dumpy

Dumpy, a membrane anchored extracellular matrix protein, the majority portion of it extending into ECM, plays an important role in the epithelial tissue attachment with adjacent ECM fibers. The protein is well anchored, through its cytoplasmic domain, in epithelial tissue enabling it to bear the mechanical strength. 68 reported that dumpy protein, through its motif (repeated modular unit) anchors the epithelial tissue to overlying cuticle structure. It plays a critical role in maintaining the infrastructure and integrity of cuticle structure and drives the process of morphogenesis during development. In addition, dumpy protein also involved in wing morphogenesis (68).

### 4.12 Resilin

**Figure 7:**
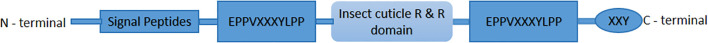
The block and domain arrangements of the resilin cuticle protein.

Resilins are elastomeric proteins that were first identified in the *D. melanogaster* (36). Weis-Fogh (69) discovered a rubber-like protein within the ECM of the thorax, wings, and hinge ligaments of dragonflies and locusts, designated them as resilin proteins. These proteins are enriched with glycine and proline and are cross-linked with di-and tri-tyrosine residues which provide them mechanical elasticity. The resilin sequence contains two isoforms called pro-resilins isoform A and isoform B. Ardell and Andersen (36)Lyons et al. (70) concluded that *Drosophila melanogaster*, Flea and Dragon fly have EPPVXXXYLPP consensus residues after a signal peptide at the N-terminal and GYSGGRPGGQDLG consensus sequence present before the C-terminal. This family proteins also consists of RR-2 conserved regions in the complete protein sequence. A tyrosine residue present at C - terminus and YGAP residues present at N- terminus in the resilin block arrangements **Figure 7**). Tyrosine residues function to cross-link with ECM (36). The RR sequence is responsible for the binding of resilin to the cuticle.

#### 4.12.1 Function of resilin

Resilins are present in high amounts in the joints and hinges of insects, where they provide elasticity and resiliency to these structures and ward off muscle fatigueness (71). Resilins are capable of storing kinetic energy, providing high resilience, low stiffness and large strain in the tendons of insects, attributing to efficient movement and locomotion (72). All the arginine and lysine residues in the peptide chain are followed by a proline residue, which makes the peptide bond resistant to trypsin hydrolysis (36).

### 4.13 CPTC family

*An. sinensis* is also frequently discovered in dipterans, and the name implies that the cuticle protein has the two conserved cysteines that share the cysteine-containing CBDs and determined four CPTCs (CPTC 1-4) in *An. gambiae* (22, 73). The same sequence is found in the gene *GASP* (Gene Analogous to Small Peritrophins), which is related to the *Drosophila* cys-rich gene and is located in the tracheal epidermis (22).

#### 4.13.1 Function of CPTC

According to Adams et al. (74), CPTC proteins are involved in the chitin-binding cuticle formation process. The CPTC functions as a serine protease inhibitor and provide protection to the old cuticle from the proteases activities. The CPTC family plays a major role in tracheae during the metamorphosis and development (73).

## 5 Role of cuticle in mosquitoes

**Figure 8:**
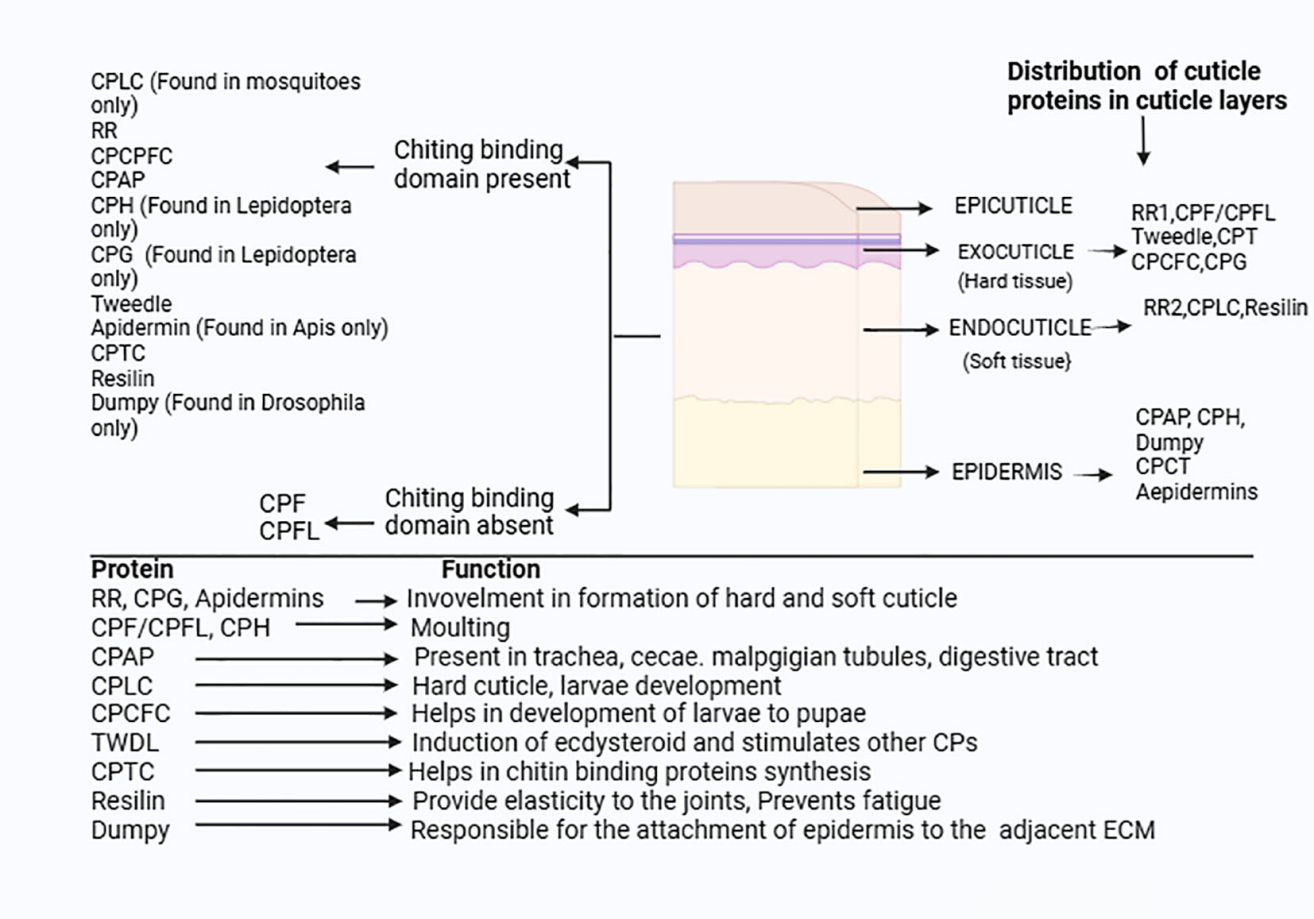
Distribution and function of CPs in insects: The CPs families are divided into the two major group based on presence and absence of the chitin binding domain.

Beyond maintaining structural integrity, CPs in mosquitoes also affect physiological and life history behavioral traits that are essential for survival, adaptability and reproduction. This section discusses about emerging role of cuticle proteins, particularly focus on their implication in IR, mating biology and developmental stage-specific functions (**Figure 8**).

### 5.1 Role in insecticide resistance

The use of insecticide is one of the first and most efficient methods for vector control in order to combat VBD. However, prolonged and over use of these chemical toxins designed against mosquito vectors (MVs) led to development of resistance. There are several mechanisms such as genetic, metabolic, behavioral, microbial, physiological and penetration resistance through which MVs have acquired resistance against conventionally used insecticide.

Contact dependent insecticide such as pyrethroid must traverse the cuticle before reaching their target, nervous system. Mosquitoes like other insects have developed penetration resistance mechanisms, to reduce the insecticide penetration. The cuticle modification in *An. gambiae* and *Ae. aegypti* is considered one of the key mechanisms conferring insecticide resistance (IR). Cuticular resistance is far less well known than other IR mechanisms such as CYP enzyme mediated detoxification and Knockdown resistance (kdr). The emerging evidence demonstrates that CPs attributes to insecticide resistance primarily by altering the structural and biochemical properties of the mosquito cuticle, thereby preventing permeability to insecticides. One key strategy is thickening of cuticle layers, facilitated by enhanced expression of certain CPs, like CPLCG3, identified in resistant *An. gambiae*. (75). In *Culex pipiens pallens*, the *CPLCG5* gene is differentially expressed in mosquito legs which reduces the penetration of insecticides by hardening the extracellular matrix (ECM) of the cuticle during insecticide exposure in mosquitoes. It is CPLCGs that contribute to insecticide resistance by facilitating the formation of a rigid matrix and increasing the thickness of the cuticle (76). Another study reported heightened expression of two *CPLC* genes in resistant strains of *Anopheles stephens*i compared to susceptible strain mosquitoes (77). The relatively high expression of both *CPLCG3* and *CPLCG4* genes in the appendages of *An. gambiae* indicates that they are possibly accountable for conferring resistance to pyrethroids (78). These proteins are presumed to modify the cuticle structure by increasing deposition of cuticle material, thereby reducing the permeability to contact insecticides.

In addition to cuticle thickening, alteration in the structural component of cuticle layer, such as increased content of chitin polysaccharide(structural constituent of cuticle), led to slow or reduced absorption of pyrethroids in mosquito (79). Though, cuticle alteration mechanisms attribute low level of protection but become significant when synergies with other resistance mechanisms (79–81). Yahouédo et al. (75) reported synergy of cuticle and metabolic process conferring resistance in MRS strain of *An. gambiae* (lack kdr mutation). The overexpression of cuticle genes leads to thickening of the cuticle layer which slows the permeation of insecticide and culminates in extended time for metabolic enzymes to detoxify and excrete out toxins.

A study on *Anopheles albimanus* also highlight that insecticide exposure changes the bacterial diversity on both cuticle surface and internal tissues, promoting selection and abundance of *Pantoea agglomerans* and *Pseudomonas fragi*, well known insecticide metabolizing enzyme, thus attributing to IR (82, 83). This study demonstrates the possible link of host symbiotic bacteria with insecticide resistance.

However, the regulatory molecular mechanism governing the *CP* gene expression during IR remains poorly explored demonstrating the need for future research at molecular and functional level.

### 5.2 Role in mating behavior

In addition to maintaining structural functions, the cuticle hydrocarbons associated with CPs also play a vital role in sexual communication in mosquitoes. Understanding how these compounds influence mating dynamics offers insights into vector population maintenance and opportunities for novel control strategies. A study in *Anopheles coluzzii* reported increased cuticle hydrocarbons in mated male compared to unmated male reflect the possible role of CHC in the mating behavior (84). Furthermore, Wang et al. (85), study demonstrated that when *Anopheles stephensi* male was treated with cuticle hydrocarbon heptacosane, it led to increased rate of insemination compared to untreated male highlighting its role in sexual behavior. These findings suggest the importance of cuticle associated hydrocarbons in mosquito reproductive success, which may be leveraged to develop mating behavior intervention vector control strategies.

### 5.3 Stage-specific functions

CPs are differentially expressed in stage-specific manner, facilitating mosquitoes to adapt in different environmental conditions at each phase of its life cycle. In *Aedes aegypti*, CPR100, a key constituent of the cuticle layer in the procuticle is essential for survival, egg development and hatching during aquatic developmental stages. Knockdown of the CPR100 and/or its associated protein result in mortality, reduced egg hatching rate, impaired egg shell formation and enhanced sensitivity to low temperature. (86). These findings highlight that CPs also contribute in developmental transition of mosquito not only structural components but also play active roles in regulating developmental transitions.

Given that CPs cater to a wide range of functions, further exploring the role of CPs and its associated auxiliary protein catering to different physiological processes could reveal potential targets to control mosquito vectors and VBDs.

## 6 Research gap

1. The CPFL/CPF Protein Family, which was first discovered in lepidopterans, is still poorly understood in other insect orders. There is currently very few research available. Similarly, the CPCFC protein family has also been least explored across different arthropods species. The existing research on CPFL/CPF and CPCFC is confined to specific insect species, leaving a knowledge gap about their role characterization and distribution across other insect species. Understanding the physiological roles of these underexplored cuticle protein genes families may unravel new molecular targets for disrupting pest or mosquito cuticle integrity.
2. The cuticle proteins such as dumpy, tweedle, resilin, and CPH have been characterized only in model organisms such as *Drosophila melanogaster* and *Bombyx mor*i. Their role needs to be explored in other insects. Their functional roles need to be explored in mosquito vector species, especially *Anopheles* and *Aedes*. Further deciphering their functional role in vector mosquitoes may unravel vector-specific functions that may be exploited in targeted vector control strategies.
3. The RR families cuticle protein and resilins proteins share consensus RR cuticle protein domains with few amino acids differences. Not enough studies have been conducted to determine the evolutionary link between the RR family and resilin, including their origin and diversion or possible categorization of resilins as a subgroup within the RR family.
4. Microbiota directly or indirectly through its metabolites via gut- brain-axis affect the wide array of insect biology including survival, development, immunity and cuticle formation (87). Insecticide exposure changes the bacterial community inclusive of cuticle surface bacteria. However, how this alteration in bacterial communities influence the metabolite profile and how this altered metabolite affects the cuticle surface- its structure, composition and resistance is not explored. This information gap emphasizes the importance of further inquiry into how altered bacterial populations affect metabolite profile and sequentially structure of cuticle and IR mechanism and thereby knowledge could be leveraged to reduced cuticle mediated resistance in vector mosquitoes.
5. Different MVs inhabiting different geography exhibit specific cuticle compositions that influence IR. Identifying common CPs providing resistance may reveal a shared cuticle resistance mechanism. Identifying common CPs associated with cuticle resistance may aid in developing molecular markers to detect cuticle mediated resistance.
6. Insect employs a multifaceted approach including genetic, behavioral, physiological and cuticle resistance to neutralize insecticide toxicity. While each strategy has been examined independently, there is a need to explore whether there is a regulatory mechanism that synchronizes all these processes to work in tandem. This lack of knowledge limits our understanding on interplay of cuticle resistance with different resistance mechanisms. Exploring this mechanistic interaction of cuticle resistance with different resistance mechanisms may reveal new molecular targets that can be leveraged to reduce the overall resistance mechanism.
7. The CPs offer a multitude of functions across diverse insect species ranging from structural support, contributing to immunity, preventing severe environmental stress and insecticides, to facilitating development essential for their survival and development. Although, role of different CPs have been decoded in insects and mosquitoes, leaving a significant knowledge gap on molecular signaling pathway, regulatory process involved in their gene expression and the molecular cross talk of CPs with other metabolic networks to drive any insect behavior remains unexplored.

The role of CPs goes beyond just being a structural protein. We emphasized the necessity of further research to unravel the underlying molecular intricacies of CPs in order to narrow down the current knowledge gap in this vital field of insect biology. Addressing these research gaps might open new avenues for effective pest and vector control.

## 7 Conclusions

This review focuses on providing comprehensive information on cuticle proteins and emphasizes their importance in insect and mosquito vectors, especially *Anopheles.* The diversity of cuticular proteins across different insect species reflects adaptations to various ecological niches and environmental conditions. Investigating the diversity, expression patterns, and functions of cuticular proteins can provide insights into the evolutionary processes that shaped insect morphology and physiology. With the rapid emergence of insecticide resistance presenting a significant threat to pest and vector control strategies, it is imperative to develop novel innovative strategic initiatives for sustainable pest and vector control management.

The majority of research studies have just focused on comprehending the role of CP, leaving a research gap on molecular mechanisms. Future research deciphering regulatory pathways, molecular signaling and molecular cross-with metabolic pathways may reveal new molecular target for gene based interventions like CRISPR-mediated knockdowns or RNA silencing, also known as RNA interference (RNAi). Furthermore, integration of these molecular mechanistic insights with advanced genomic and bioinformatic tools might unravel the key molecular targets for developing species specific vector or insect pest control strategies.
